# Sleep structure and related clinical characteristics in drug-naïve Parkinson's disease with subjectively different sleep quality

**DOI:** 10.3389/fneur.2023.1156910

**Published:** 2023-05-31

**Authors:** Yinyin Jiang, Yaning Chen, Dongfeng Li, Sha Zhu, Ruxin Gu, Yaxi Wang, Jun Zhu, Xu Jiang, Bo Shen, Yang Pan, Jun Yan, Li Zhang

**Affiliations:** ^1^Department of Geriatric Neurology, Nanjing Brain Hospital Affiliated to Nanjing Medical University, Nanjing, China; ^2^Institute of Neuropsychiatric Diseases, Nanjing Brain Hospital Affiliated to Nanjing Medical University, Nanjing, China

**Keywords:** Parkinson's disease, sleep disturbances, non-motor symptoms, polysomnography, sleep structure, clinical features

## Abstract

**Background:**

Sleep disturbance is a common non-motor symptom of Parkinson's disease (PD). Most polysomnography (PSG) studies are conducted when patients are in their “on medication” state. Our study aimed to investigate changes in the sleep structure in drug-naive PD patients with poor subjective sleep quality based on polysomnography (PSG) and to explore potential correlations between sleep structure and clinical features of the disease.

**Methods:**

A total of 44 drug-naive PD patients were included. All patients completed a standardized questionnaire to obtain demographic and clinical characteristics and underwent whole-night PSG recording. Patients with PSQI scores >5.5 were considered poor sleepers, and patients with PSQI scores <5.5 were considered to be good sleepers.

**Results:**

There were 24 (54.5%) PD patients in the good sleeper group and 20 (24.5%) PD patients in the poor sleeper group. We observed that poor sleepers had severe non-motor symptoms (NMS) and worse life quality. The PSG displayed that they had a longer wake-up time after sleep onset (WASO) and lower sleep efficiency (SE). Correlation analysis revealed that the micro-arousal index was positively associated with UPDRS-III, and the N1 sleep percentage was negatively associated with the NMS score in good sleepers. For poor sleepers, rapid eye movement (REM) sleep percentage was negatively related to the Hoehn-Yahr (H-Y) stage, WASO increased with UPDRS-III, periodic limb movement index (PLMI) increased with the NMS score, and N2 sleep percentage was negatively related to the score of life quality.

**Conclusion:**

Night awakening is the main manifestation of decreased sleep quality in drug-naive PD patients. Poor sleepers have severe non-motor symptoms and poor life quality. Additionally, the increase in nocturnal arousal events may predict the progression of motor dysfunction.

## 1. Introduction

Parkinson's disease (PD) is a common chronic neurodegenerative disease that places an enormous material and mental burden on patients (1, 2). Although PD is characterized by motor symptoms, a broad spectrum of non-motor symptoms (NMS), including sleep disturbance, neuropsychiatric symptoms, and autonomic dysfunction, can be identified in people with PD (3). Sleep disturbances in PD include insomnia, rapid eye movement (REM) sleep behavior disorder, sleep-disordered breathing, restless legs syndrome, and circadian dysregulation, and affect a high percentage of people with PD (4, 5). The decline in sleep quality is a very common complaint among PD patients. One possible reason for the high prevalence of sleep complaints is that neurodegenerative diseases disrupt the circadian rhythm system at a faster rate than normal aging (6). Furthermore, there are strong correlations between sleep disturbances and other non-motor as well as motor symptoms such as nocturia, dystonia, and pain (7). Worse sleep quality has recently been reported to aggravate motor and non-motor symptoms in PD (8, 9). Many sleep problems may occur in the early stages of the disease and worsen as the disease progresses (10, 11), necessitating proper vigilance.

In the past, the evaluation of sleep quality relied mainly on a series of sleep scales. Many studies have used the Pittsburgh Sleep Quality Index (PSQI) and the Parkinson's Disease Sleep Scale (PDSS) for sleep assessment in PD patients (12). Although these instruments are rapidly administered and efficient, a detailed understanding of the sleep structure of patients with sleep complaints is still lacking. Currently, video-polysomnography (v-PSG), which is considered to be the most valuable method of diagnosing sleep disorders, is widely applied (13). PSG can not only quantitatively analyze the sleep structure but can also sensitively record the occurrence of specific sleep events. Previous studies using PSG revealed that, compared with healthy controls, patients with PD have shorter total sleep time, lower sleep efficiency, and increased sleep latency and wakefulness after sleep onset (14–16). However, anti-PD medication has an inevitable effect on sleep structures. The use of MAO-B inhibitors is related to sleep stages 2 and 3, while sleep stage 1 can be predicted by dopamine agonists (17).

In this respect, a cohort of drug-naive patients is essential to bringing new insights into the field of the non-motor spectrum of PD. This study aimed to investigate changes in the sleep structure in drug-naive PD patients with poor subjective sleep quality compared to those with good sleep quality using PSG technology and to explore the association between clinical features and sleep structure.

## 2. Materials and methods

### 2.1. Study participants

A total of 63 newly diagnosed PD patients were initially recruited from the Neurology Clinic of the Affiliated Brain Hospital of Nanjing Medical University from September 2021 to May 2022. Patients were diagnosed by at least two movement disorder disease specialists. Participants who met the following criteria were eligible for inclusion: if they met the diagnostic criteria of the UK Parkinson's Disease Brain Bank (18) and did not use any anti-PD medication before if they completed a standard questionnaire completely, or if they also cooperatively performed a PSG examination before taking any anti-PD medication. Exclusion criteria were as follows: (1) the presence of secondary parkinsonism, focal brain lesions (according to MRI evidence), and other psychiatric disorders; (2) patients taking hypnotics, antidepressants, or antipsychotics within the month prior to the PSG examination, because these drugs may affect sleep architecture; and (3) the presence of severe obstructive sleep apnea (apnea–hypopnea index was higher than 30), in which situation, there was manual intervention during the PSG, which could affect the reliability of the PSG. Finally, 44 patients were included in this study. This study was approved by the Ethics Committee of the Affiliated Brain Hospital of Nanjing Medical University (2021-KY007-01), and written informed consent was obtained from the study participants for research purposes.

### 2.2. Clinical evaluation

All subjects completed a standardized questionnaire designed to collect general information, including name, age, gender, body mass index (BMI), and disease duration at baseline. The Unified Parkinson's Disease Rating Scale (UPDRS) (19) and their Hoehn -Yahr stage (H-Y stage) (20) were used to assess comprehensive symptoms and to describe the severity of motor function. Non-motor symptoms were assessed using the Non-Motor Symptom Questionnaire (NMSQ) (21, 22). The Montreal Cognitive Assessment (MoCA) was used to evaluate cognitive function (23). Quality of life was assessed using the 39-item Parkinson's Disease Questionnaire (PDQ-39) (24). Subjective sleep assessment was performed using the Pittsburgh Sleep Quality Index (PSQI) (25) and the Parkinson's Disease Sleep Scale (PDSS) (26). The diagnosis of rapid eye movement behavior disorder (RBD), periodic limb movement disorder, and obstructive sleep apnea was made based on the PSG reports according to the International Classification of Sleep Disorders, Third Edition criteria (27). The PSQI is a self-report questionnaire that evaluates the subject's sleep during the past month. It includes 19 items, and the maximum possible score is 21. A PSQI global score of >5 discriminates sleep disturbance in patients with insomnia with a sensitivity of 99% and a specificity of 84% (28). The cutoff value to differentiate between good and poor sleepers is >5 points (25). We considered patients with PSQI scores >5.5 as poor sleepers and patients with PSQI scores < 5.5 to be good sleepers.

### 2.3. Polysomnography technique

All patients underwent overnight video-PSG in an acclimatized, sound-proof sleep laboratory with a data acquisition time longer than 8 h. We used a Compumedics Grael-HD 64 (Compumedics Grael-HD, Melbourne, Australia) polygraph for signal acquisition. This instrument includes six electroencephalogram channels, two electrooculogram channels, one electromyogram channel, one nasal pressure channel, one respiratory thermistor channel, one oximetry channel, one snore detector channel, one electrocardiography channel, one abdominal movement assessment channel, one thoracic movement assessment channel, one body position channel, and two leg movement sensors. Continuous audio and video recording were performed using infrared cameras. The EEG channels, including frontal (F3, F4), central (C3, C4), and occipital (O1, O2) channels, were placed according to the international 10–20 location system and linked to the mastoid electrodes as a reference. The staging and scoring were reviewed by a board-certified sleep physician according to the American Academy of Sleep Medicine (AASM) guidelines (13). Sleep stage percentages were calculated relative to total sleep time. Objective sleep parameters included total sleep time (TST; min), wake-up time after sleep onset (WASO; min), sleep efficiency (SE; %), sleep latency (SL; min), and the percentage of N1, N2, and N3 sleep, average heart rate (AHR; the number of events/min sleep), apnea–hypopnea index (number of events/hour sleep), micro-arousal index (number of events/hour sleep), and periodic limb movement index (PLMI; the total number of leg movements) during the entire night.

### 2.4. Statistical analysis

IBM SPSS 26.0 software was used for statistical analysis. Measurement data were expressed as mean (standard deviation, SD), median (interquartile range, IQR), number (N), or percentage (%). The Kolmogorov–Smirnov test was used to check the normality of the measurement data. A two-sample *t*-test was used for normally distributed data, and the Mann–Whitney *U*-test was used for non-normally distributed data. A chi-square test was conducted to analyze qualitative data. PSG data were compared by one-way analysis of covariance (ANCOVA) with adjustments for confounding factors, including UPDRS-III score and H-Y stage. In addition, the Spearman test was applied to analyze the correlation between sleep parameters and clinical characteristics. Linear regression was performed with the evaluation of the correlation coefficient. The statistical significance level was set at a *p* < 0.05.

## 3. Results

### 3.1. Demographic and clinical characteristics

Table 1 shows the demographic information and clinical characteristics of the patients. A total of 63 drug-naive PD patients were initially examined. A total of 13 patients who took hypnotics, antidepressants, or antipsychotics within the month prior to the PSG examination were excluded. Four patients were excluded due to incomplete PSG data, and two patients who had severe obstructive sleep apnea were also excluded. Finally, 44 patients were included in our study. Among them, 24 (54.5%) were good sleepers, and 20 (45.5%) were poor sleepers. Compared with good sleepers, poor sleepers had higher NMSQ and PDQ-39 scores and lower PDSS scores (*P* = 0.026, *P* = 0.010, *P* = 0.001, respectively). However, there were no significant differences in age, sex, BMI, disease duration, H-Y stage, UPDRS-III, or MoCA scores between the two groups. Furthermore, we observed that 36.4% of our PD cohort had RBD, 27.3% had PLMD, and 27.3% had OSA. We compared the distribution of various sleep disorders in good and poor sleepers, and no significant difference was found.

**Table 1:** Demographic information and clinical characteristics.

**Variables**	**Total**	**Good sleepers (N=24)**	**Poor sleepers (N=20)**	***P***
Age, years	68.86 (7.06)	68.83 (8.67)	68.90 (4.93)	0.983^a^
Male patients/female patients, N	30/14	18/6	12/8	0.652^c^
BMI, kg/m^2^	24.48 (3.06)	24.94 (2.60)	23.94 (3.60)	0.458^a^
Disease duration, years	1.25 (3.5)	1 (2.5)	2 (3.4)	0.172^b^
H-Y stage	2 (0)	2 (0)	2 (0.1)	0.398^b^
UPDRS III	19.73 (6.94)	18.08 (7.43)	21.70 (6.08)	0.232^a^
NMSQ	8.68 (4.49)	7.50 (4.40)	10.10 (4.38)	**0.026** ^**a**^
MoCA	27 (4)	25 (5)	27 (1)	0.130^b^
PDSS	112 (40)	132 (17)	94.5 (13)	**0.001** ^**b**^
PDQ-39	15.50 (41)	10 (21)	42.5 (38)	**0.010** ^**b**^
Sleep disorders, N (%)				
RBD	16 (36.4%)	10	6	0.675^c^
PLMD	12 (27.3%)	8	4	0.646^c^
OSA	12 (27.3%)	10	2	0.162^c^

^a^Student's t-test, ^b^Mann–Whitney U-test, ^c^chi-square test. Data with a superscript a are expressed as mean (standard deviation, SD), those with a superscript b as median (interquartile range, IQR), and those with a superscript c as number (percentage). Bold values indicate statistically significant differences (p < 0.05).

BMI, body mass index; H-Y stage, Hoehn–Yahr stage; UPDRS-III, Unified Parkinson's Disease Rating Scale Part III; NMSQ, Non-motor Symptoms Questionnaire; MoCA, Montreal Cognitive Assessment; PDSS, Parkinson's Disease Sleep Scale; PDQ-39, Parkinson's Disease Questionnaire-39; RBD, rapid eye movement sleep behavior disorder; PLMD, periodic limb movement disorder; OSA, obstructive sleep apnea.

### 3.2. Comparison of sleep scales

The scores of patients with and without various sleep disorders on two sleep scales were compared separately; the results are summarized in Table 2. No significant differences were found for RBD, PLMD, or OSA patients.

**Table 2:** Comparison of sleep scales between different sleep disorders.

	Without RBD	With RBD	*P*
PSQI	4 (6)	2 (7)	0.583^b^
PDSS	110.69 (22.33)	115.56 (24.45)	0.995^a^
			
	Without PLMD	With PLMD	*P*
PSQI	6.17 (4.81)	4.71 (3.52)	0.654^a^
PDSS	111 (24.1)	118.93 (18.71)	0.693^a^
			
	Without OSA	With OSA	*P*
PSQI	6.63 (4.62)	4.43 (4.24)	0.148^a^
PDSS	110.88 (20.89)	116.04 (27.1)	0.084^a^

^a^Student's t-test, ^b^Mann–Whitney U-test. Variables with a superscript a are expressed as mean (standard deviation, SD) and those with a superscript b as median (interquartile range, IQR). Bold values indicate statistically significant differences (p < 0.05).

PSQI, Pittsburgh Sleep Quality Index; PDSS, Parkinson's Disease Sleep Scale; RBD, rapid eye movement sleep behavior disorder; PLMD, periodic limb movement disorder; OSA, obstructive sleep apnea.

### 3.3. Comparison of sleep parameters

Sleep parameters derived from PSG recordings are shown in Table 3. Poor sleepers differed significantly from good sleepers in terms of wake-up time after onset (WASO) and sleep efficiency (SE) (*P* = 0.008, *P* = 0.004, repetitively). There was no significant difference between the groups in total sleep time, sleep latency, REM latency, N1 sleep percentage, N2 sleep percentage, N3 sleep percentage, or the sleep disorder indices average heart rate (AHR), apnea–hypopnea index (AHI), micro-arousal index (MAI), and periodic limb movement index (PLMI).

**Table 3:** Comparison of sleep parameters.

	**Total**	**Good sleepers**	**Poor sleepers**	***P***
TST (SD), min	308.50 (110.70)	339.29 (99.66)	271.55 (116.94)	0.158^a^
WASO (SD), min	171.00 (79.12)	144.75 (49.87)	199.00 (64.75)	**0.008** ^**b**^
SE, %	53.84 (16.67)	58.18 (14.74)	48.64 (18.10)	**0.004** ^**a**^
SL(SD), min	23.00 (32.3)	27.50 (45.6)	23.00 (56.9)	0.974^b^
REML (SD), min	116.50 (198.5)	102.50 (88.0)	164.00 (349.3)	0.470^b^
REM Sleep% (SD), min	12.01 (8.25)	12.54 (9.67)	11.38 (6.61)	0.751^a^
N1 Sleep %	6.65 (12.30)	5.80 (12.30)	9.05 (12.78)	0.817^b^
N2 Sleep %	62.84 (12.43)	63.82 (13.30)	61.67 (11.90)	0.694^a^
N3 Sleep %	13.45 (27.50)	9.55 (27.05)	21.85 (22.28)	0.322^b^
AHR (SD)	60.39 (7.15)	59.88 (7.74)	61.00 (6.72)	0.725^a^
AH I(SD)	1.90 (7.0)	3.95 (8.0)	0.35 (3.3)	0.137^b^
MAI (SD)	12.95 (8.5)	15.80 (5.4)	9.7 (9.3)	0.974^b^
PLMI (SD)	31.25 (59.98)	47.71 (78.20)	11.50 (11.24)	0.459^a^

^a^Student's t-test, ^b^Mann–Whitney U-test. Variables with a superscript a are expressed as mean (standard deviation, SD) and those with a superscript b as median (interquartile range, IQR). Bold values indicate statistically significant differences (p < 0.05). The p-value was adjusted for the UPDRS-III score and H-Y stage.

TST, total sleep time, WASO, wake time after sleep onset, SE, sleep efficiency, SL, sleep latency, REM, rapid eye movement, REML, REM sleep latency, N1 sleep, NREM sleep stage 1, N2 sleep, NREM sleep stage 2, N3 sleep, NREM sleep stage 3, AHR, average heart rate, AHI, apnea–hypopnea index, MAI, micro-arousal index, PLMI, periodic limb movement index.

### 3.4. Correlations between sleep architecture and clinical features

The correlations between sleep parameters and clinical features are presented in Table 4. For good sleepers, increasing MAI was related to higher UPDRS-III scores (r = 0.680, *P* = 0.015), and N1 sleep percentage increased with NMSQ scores (r = −0.564, *P* = 0.036). For poor sleepers, longer WASO was associated with higher UPDRS-III scores (r = 0.640, P = 0.006), PLMI increased with NMSQ scores (r = 0.644, *P* = 0.014), and lower N2 sleep percentage was related to higher PDQ-39 scores (r = −0.685, *P* = 0.029). We also observed that REM sleep latency increased with the H-Y stage in poor sleepers (r = −0.696, *P* = 0.025).

**Table 4:** Correlations between different sleep parameters and clinical features.

	**Good sleepers**							
	**H-Y stage**		**UPDRS-III**		**NMSQ score**		**PDQ-39**	
	**r**	***p***	**r**	***p***	**r**	***p***	**r**	***p***
TST	0.063	0.846	0.088	0.786	−0.091	0.778	0.174	0.589
WASO	0.209	0.514	−0.049	0.880	−0.361	0.249	−0.298	0.346
SE	0.000	1.000	0.175	0.586	−0.086	0.791	0.204	0.526
SL	0.000	1.000	−0.221	0.491	0.264	0.406	0.021	0.948
REML	−0.238	0.480	−0.478	0.128	0.121	0.722	−0.064	0.852
REM Sleep%	0.042	0.897	0.530	0.058	−0.007	0.982	0.119	0.712
N1 Sleep %	0.376	0.228	0.070	0.829	**−0.564**	**0.036**	−0.396	0.202
N2 Sleep %	−0.167	0.603	0.088	0.787	−0.236	0.461	−0.088	0.786
N3 Sleep %	−0.168	0.603	−0.309	0.329	0.564	0.056	0.446	0.146
AHR	−0.376	0.228	−0.553	0.062	−0.472	0.077	−0.533	0.074
AHI	−0.021	0.948	0.268	0.399	−0.268	0.399	−0.308	0.331
MAI	0.125	0.698	**0.680**	**0.015**	0.043	0.895	0.270	0.396
PLMI	−0.042	0.897	0.172	0.594	0.134	0.320	−0.053	0.871
	**Poor sleepers**							
	**H-Y stage**		**UPDRS-III**		**NMSQ score**		**PDQ-39 score**	
	**r**	***p***	**r**	***p***	**r**	***p***	**r**	***P***
TST	−0.609	0.062	−0.293	0.412	−0.301	0.399	0.006	0.987
WASO	0.609	0.062	**0.640**	**0.006**	0.153	0.672	−0.067	0.855
SE	−0.609	0.062	−0.524	0.120	−0.129	0.723	−0.067	0.855
SL	0.000	1.000	0.390	0.265	−0.288	0.419	−0.176	0.627
REML	0.274	0.476	0.151	0.699	−0.201	0.604	−0.283	0.460
REM Sleep%	**−0.696**	**0.025**	−0.354	0.316	−0.166	0.647	0.382	0.276
N1 Sleep %	−0.087	0.811	−0.134	0.712	0.522	0.122	0.442	0.200
N2 Sleep %	0.348	0.324	0.317	0.372	−0.313	0.379	**−0.685**	**0.029**
N3 Sleep %	0.087	0.811	−0.152	0.674	0.252	0.483	0.212	0.556
AHR	−0.225	0.533	0.567	0.055	0.070	0.848	0.600	0.067
AHI	0.301	0.064	−0.043	0.043	−0.488	0.153	0.524	0.120
MAI	−0.522	0.122	0.232	0.519	0.544	0.054	0.067	0.855
PLMI	0.435	0.209	0.134	0.712	**0.644**	**0.014**	0.164	0.651

Bold values indicate statistically significant differences (p < 0.05).

H-Y stage, Hoehn–Yahr stage; UPDRS-III, Unified Parkinson's Disease Rating Scale Part III; NMSQ, Non-motor Symptoms Questionnaire; PDQ-39, Parkinson's Disease Questionnaire-39; TST, total sleep time, WASO, wake time after sleep onset, SE, sleep efficiency, SL, sleep latency, REM, rapid eye movement, REML, REM sleep latency, N1 sleep, NREM sleep stage 1, N2 sleep, NREM sleep stage 2, N3 sleep, NREM sleep stage 3, AHR, average heart rate, AHI, apnea–hypopnea index, MAI, micro-arousal index, PLMI, periodic limb movement index.

The regression analysis between clinical features and associated sleep parameters is summarized in Table 5. In good sleepers, MAI explained 46.8% of UPDRS-III variation (*P* = 0.003, R^2^ = 0.468), and N1 sleep percentage explained 33.4% of NMSQ variation. For patients with poor sleep quality, PLMI accounted for 31.6% of NMSQ variation (*P* = 0.033, R^2^ = 0.316), and N2 sleep percentage accounted for 36.7% of PDQ-39 variation (*P*= 0.037, R^2^ = 0.367).

**Table 5:** Linear regression analysis in good sleepers and in poor sleepers.

	**B**	**β**	**t**	***p***	**F**	**Adjusted R^2^**
**Good sleepers**						
UPDRS-III						
MAI	0.897	0.718	3.265	0.009	10.660	0.468
NMSQ score						
N1%	−0.319	−0.628	−2.554	0.029	6.522	0.334
						
**Poor sleepers**						
NMSQ score						
PLMI	0.244	0.626	2.272	0.013	5.163	0.316
PDQ-39 score						
N2%	−1.106	0.444	−0.661	0.037	6.210	0.367

The p-value has been calculated using a linear logistic regression analysis.

UPDRS-III, Unified Parkinson's Disease Rating Scale Part III; NMSQ, Non-motor Symptoms Questionnaire; PDQ-39, Parkinson's Disease Questionnaire-39; N1 sleep, NREM sleep stage 1, N2 sleep, NREM sleep stage 2, MAI, micro-arousal index, PLMI, periodic limb movement index.

## 4. Discussion

A total of 44 patients were enrolled in this study, including 24 (54.5%) in the good sleeper group and 20 (45.5%) in the poor sleeper group. We observed that poor sleepers had severe non-motor symptoms and poor quality of life. They had longer wake-up times and lower sleep efficiency during night sleep according to PSG results. Furthermore, we found significant associations between nocturnal arousal events and motor symptom scores in all drug-naive PD patients. Our findings provide new insights into sleep problems in Parkinson's disease.

Our study evaluated the prevalence of three major sleep disorders in PD, including RBD (36.4%), PLMD (27.3%), and OSA (27.3%). The results are consistent with the frequencies reported in previous studies (29, 30). We did not find significant differences in PSQI or PDSS scores between the groups with and without these three sleep disorders. The results are consistent with those reported in some studies (31, 32), but contrary to other studies (33, 34). Differences between the results obtained in this study and other studies may be explained by the possible coexistence of other sleep problems in different PD cohorts and by the fact that only one or two questions on the PSQI and PDSS scales are strongly associated with specific types of sleep disorders. A previous study that tested the criterion validity of the PSQI in diagnosing the sleep disorders mentioned above found that it was not highly accurate for predicting the existence of these disorders (35). Thus, the PSQI is more suitable for screening for the presence of sleep alterations than for diagnosing specific types of sleep disorders. In our study, we divided the patients into two groups based on their PSQI scores. We compared the distribution of sleep disorders in good and poor sleepers, and no significant difference was found.

Changes in PSG in our study focused on drug-naive PD patients. After considering the distribution of different sleep disorders, we wanted to investigate the features of disturbed objective sleep parameters in patients suffering from reduced subjective sleep quality. Previous studies have reported that, compared with healthy controls, there were significant differences in sleep architecture patterns in PD patients (16); these differences include a decrease in TST, SE, N2 sleep percentage, and REM sleep percentage and an increase in WASO, N1 sleep percentage, REM sleep latency, and PLMI. Our study compared the sleep structure between good and poor sleepers in drug-naive patients. We observed that as patients' sleep quality began to decline, the main changes were longer wake-up times after falling asleep and lower sleep efficiency. The differences in the findings may be explained by the progression of the disease and the use of anti-PD medication. In terms of neuropathologic evidence, sleep–wake disturbances in PD may reflect disruption of the neural circuitry that controls circadian rhythms (6). Altered peripheral clock gene expression and decreased function of the hypothalamic suprachiasmatic nucleus (SCN) are believed to be responsible for reduced melatonin output and sleep–wake disruption in PD patients (36, 37). Our results also revealed significant associations between the percentage of REM sleep and the H-Y stage in poor sleepers. Sleep-controlling neurons have recently been reported to be sensitive and vulnerable to α-synuclein (38), and the spread of pathology to the coeruleus/subcoeruleus complex may further disrupt REM sleep (39–41). With regard to anti-PD medication, one study has reported that higher amounts of dopaminergic medications, especially L-dopa, were related to sleep fragmentation and insomnia (42). These changes have been described as a progressive destructuring of the sleep structure (43). The mechanisms underlying sleep disturbances in PD remain speculative. Our observations may be an early objective manifestation of sleep disturbance in original ecological patients.

We consider sleep disturbances an integral part of PD rather than comorbidity. Our research explored the association between sleep parameters and typical clinical characteristics of PD, including the H-Y stage, the UPDRS-III score, the NMSQ score, and the PDQ-39 score. We also conducted an analysis of correlations between disease and PSG parameters, and no significant correlations were found. However, we observed that MAI was positively related to the UPDRS-III score in good sleepers and that WASO increased with the UPDRS-III score in poor sleepers. Both MAI and WASO were nocturnal arousal events, and dopamine may play a crucial role in the close association between these events and motor dysfunction. As we all know, PD is characterized by a progressive loss of dopaminergic neurons in the substantia nigra, and its metabolism and activity are also strongly influenced by the circadian clock (44). On the contrary, sleep dysfunction can disrupt the circadian rhythms that are generated by SCN, and the dysfunction of sleep and peripheral clocks, such as those in microglial and neuronal cells, leads to subsequent changes within and outside of the brain, including neuroinflammation, increased oxidative stress, and reduced metabolic clearance. These outcomes have been proposed to exacerbate Parkinson's disease progression (45–48). Therefore, the increase in nocturnal arousal events may be of great value in predicting the progression of motor dysfunction in PD. Our recent study found that N3 sleep (slow-wave sleep, SWS) is one of the important risk factors in PD patients with hallucinations (49). The deconstruction of the sleep structure is a process affected by many complex factors. The decrease in light sleep (including N1 sleep and N2 sleep) in the early stages of the disease causes more daytime fatigue. This may aggravate non-motor symptoms or quality of life burden in drug-naive PD patients.

Periodic limb movements during sleep (PLMS) have been reported to be associated with greater symptom severity, more subjective sleep disturbance, and decreased quality of life in PD patients (34, 50). We did not observe significant differences in PLMI variation in drug-naive PD patients with different subjective sleep quality. However, our results revealed an association between PLMI and non-motor symptoms in drug-naive patients with poor sleep quality. Evidence has confirmed that there are common pathophysiological pathways between PLMS and PD (51). Nigrostriatal degeneration may promote PLMS, whereas dopaminergic enhancement may control PLMS. In our drug-naive PD cohort, the regression model suggested that increasing PLMI was an independent variable predicting a higher NMSQ score in poor sleepers.

Several methodological considerations should be mentioned. First, although there have been some studies investigating the characteristics of objective sleep parameters in drug-naive PD patients, we conducted a more comprehensive clinical evaluation of these patients and further explored the relationship between sleep parameters and clinical features. Second, our study was based on PSG data. The majority of published studies related to sleep problems used subjective tools to measure sleep quality, while only a few such studies used objective methods. However, patients in our study did not undergo a second PSG examination to eliminate the first-night effect, which should be considered as a methodological and ethnic bias. In addition, we did not include healthy controls with and without sleep disorders such as OSA, RBD, and PLMS. Finally, our cohort consisted of a relatively small number of patients and was recruited in a single center. Further studies should include larger cohorts and conduct longitudinal follow-ups to confirm the correlations between sleep parameters and clinical characteristics.

## 5. Conclusions

Our results showed that more than 50% of drug-naive PD patients have poor subjective sleep quality, mainly manifested by increased nocturnal awakening time and decreased sleep efficiency. Poor sleepers have severe non-motor symptoms and poor quality of life. Nocturnal arousal events, including MAI and WASO, may predict the progression of motor dysfunction. We provide new evidence for associations between sleep parameters and clinical characteristics in PD patients before they begin medication interference.

## Data availability statement

The original contributions presented in the study are included in the article/supplementary material, further inquiries can be directed to the corresponding author.

## Ethics statement

The study was approved by the Ethics Committee of the Affiliated Brain Hospital of Nanjing Medical University. All procedures were in accordance with the Declaration of Helsinki.

## Author contributions

YJ and LZ conceived and designed the study. LZ, XJ, and JY obtained the funding. YJ, SZ, RG, YW, YC, DL, JZ, XJ, BS, and YP collected the data. YJ, SZ, RG, and YP conducted the data analysis. YJ drafted the manuscript. All authors contributed to the article and approved the submitted version.
